# Progressive transcortical sensory aphasia and progressive ideational apraxia owing to temporoparietal cortical atrophy

**DOI:** 10.1186/s12883-015-0490-2

**Published:** 2015-11-11

**Authors:** Michitaka Funayama, Asuka Nakajima

**Affiliations:** Department of Neuropsychiatry, Ashikaga Red Cross Hospital, Ashikaga-City, 326-0843 Japan; Department of Rehabilitation, Ashikaga Red Cross Hospital, Ashikaga-City, Japan

**Keywords:** Logopenic variant of primary progressive aphasia, Posterior cortical atrophy, Transcortical sensory aphasia, Ideational apraxia, Conceptual apraxia

## Abstract

**Background:**

In contrast to frontotemporal lobar degeneration, atrophy of the focal posterior lateral cortex has not been thoroughly studied. Three clinical types of focal cortical atrophy have been described: 1) logopenic variant of primary progressive aphasia, which presents with impaired repetition despite normal articulation; 2) posterior cortical atrophy, which presents with prominent visuospatial deficits; and 3) primary progressive apraxia. All three clinical types are characterized by specific patterns of hypometabolism/hypoperfusion: the left posterior perisylvian area in the logopenic variant of primary progressive aphasia, bilateral parietooccipital areas in posterior cortical atrophy, and the parietal cortex in primary progressive apraxia. However, not every patient clearly fits into one of these categories.

**Case presentation:**

Here we describe two patients with atypical focal cortical presentations. They presented with a history of a few years of progressive transcortical sensory aphasia characterized by fluent output with normal grammar and syntax, normal repetition, sentence comprehension deficits, and anomia without loss of word meaning. They also presented with progressive apraxia that began at the initial stages. Some forms of posterior symptoms including acalculia, agraphia, and visuospatial deficits were also observed. Hypoperfusion was noted mainly in the left temporoparietal region, which is slightly posterior to the perisylvian area.

**Conclusions:**

Although our cases lack in CSF findings and PIB scan, these two cases and previous reports might suggest the existence of a subgroup of patients presenting with transcortical sensory aphasia, apraxia, and posterior symptoms (acalculia, agraphia, and visuospatial deficits) in the setting of Alzheimer’s disease. This subgroup may reflect the spectrum of clinical manifestations between logopenic variant of primary progressive aphasia and posterior cortical atrophy.

## Background

In contrast to frontotemporal lobar degeneration, the subtypes of which are clearly categorized into behavioral variant of frontotemporal dementia, non-fluent/agrammatic primary progressive aphasia (naPPA), and semantic variant PPA (svPPA) [1], dementias affecting the posterior lateral cortex have not been thoroughly studied. Some clinical subtypes have been described, including the following three: 1) logopenic variant of PPA (lvPPA), which presents with impaired repetition despite normal articulation [2, 3]; 2) posterior cortical atrophy, which presents with prominent visuospatial deficits [4–6]; and 3) primary progressive apraxia [7–9]. All three clinical types are characterized by specific patterns of hypometabolism: in the left posterior perisylvian area in lvPPA [2, 3], in bilateral parietooccipital areas in posterior cortical atrophy [4–6], and in the parietal cortex in primary progressive apraxia [7–9]. However, not every patient clearly fits into one of these categories. In 2013, Wicklund et al. [10] described two patients with Alzheimer’s disease who showed not only progressive aphasia but also progressive visuospatial deficits with prominent hypometabolism in the left occipitotemporal region. Likewise, Garcia-Azorin et al. [11] described two patients with Alzheimer’s disease who showed progressive aphasia with left parieto-temporo-occipital hypometabolism, suggestive of an overlapping entity between PPA and posterior cortical atrophy. We describe two patients with progressive transcortical sensory aphasia and progressive ideational apraxia along with posterior symptoms (acalculia, agraphia, and visuospatial deficits) in which the areas of hypoperfusion were mainly the left temporoparietal region.

**Table 1:** Demographics and initial linguistic and other neuropsychological assessment

	Case 1	Case 2
Age at onset, years/gender	65/M	50/M
Education (years)	12	16
Years of follow-up	5	7
Clinical Dementia Rating score, total (0–3) for the first 2 years	0.5	0.5
Sentence repetition	5 phrases	6 phrases
Non-word repetition	intact	intact
Forward digit span	6	6
Backward digit span	5	4
Confrontation naming in SLTA, % correct	25 (anomia)	65 (anomia)
Auditory single-word comprehension in SLTA, % correct	100	100
Auditory complex sentence commands comprehension in SLTA, % correct	20 (auditory comprehension deficits)	50 (auditory comprehension deficits)
Reading comprehension of complex sentence commands in SLTA, % correct	20 (reading comprehension deficits)	20 (reading comprehension deficits)
Written naming in Kanji in SLTA, % correct	20 (agraphia)	20 (agraphia)
Dictation of short sentences, % correct	0 (agraphia)	0 (agraphia)
Calculation in SLTA, % correct	15 (acalculia)	10 (acalculia)
Ideational apraxia	present	present
Visuoconstructional disorder	not found	present
Visuoperceptual disorder	not found	not found
Episodic memory deficits	found (2 years postonset)	Found (4 years postonset)

*SLTA* Standard Language Test of Aphasia

## Case presentations

Patients with degeneration and focal cortical presentation were recruited from the Cognitive Function Clinic at Ashikaga Red Cross Hospital between January 2008 and December 2012. Patients with typical focal cortical degeneration were excluded, including those with the three types of PPA (logopenic, semantic, and non-fluent/agrammatic), the behavioral variant of frontotemporal dementia, posterior cortical atrophy, and primary progressive apraxia. Two patients with a degenerating condition and unusual patterns of focal cortical presentations were included. Ethical aspects of this study were reviewed and approved by the Ashikaga Hospital Human Research Ethics Committee. Informed consent was obtained from the patients and their spouse due to their severe cognitive impairment.

### Patient 1

A 65-year-old ambidextrous man with 12 years of education began to experience progressive word-finding difficulty. He had been an office worker until he retired at age 60. He had no previous medical or psychiatric problems. He was referred to our hospital for evaluation of speech difficulties 1 year after the onset of aphasia. His laboratory results were normal, and no remarkable neurological findings were noted. Neurological examination was normal with no pyramidal, extra-pyramidal nor cerebellar signs.

Neuropsychological examination (Table 1) showed remarkable word-finding difficulty but no phonological paraphasias. Grammar, articulation, prosody, and repetition were also preserved. Conversational speech was hesitant, with word-finding pauses. The maximum length of sentence repetition was five phrases. Non-word repetition was preserved. His digit span was a maximum of six digits forward and five digits backward. Comprehension of single words was nearly intact, whereas comprehension of complex sentences was impaired. For example, although he was able to comprehend simple oral sentences such as “Touch your ankle”, he was unable to comprehend complex oral sentences such as “Touch your right ankle with your left hand”. Reading of complex sentences was also impaired as was oral comprehension. His writing was severely impaired compared with his speech, suggesting that he had agraphia itself that was independent of aphasia. His calculation was also severely compromised, and he was no longer able to perform simple additions of one figure that included carrying. On neuropsychological examination, his correct naming was 25 % on the Standard Language Test of Aphasia (SLTA) [12]. Although his comprehension of single words remained 100 %, comprehension of command sentences was only 20 %. Taken together, his aphasia was categorized as transcortical sensory aphasia with preserved repetition and word meaning. Reading comprehension of complex sentence commands was only 20 %. Written naming in Kanji was 20 % and dictation of short sentences was 0 %. His correct calculation was 15 % on SLTA.

**Fig. 1:**
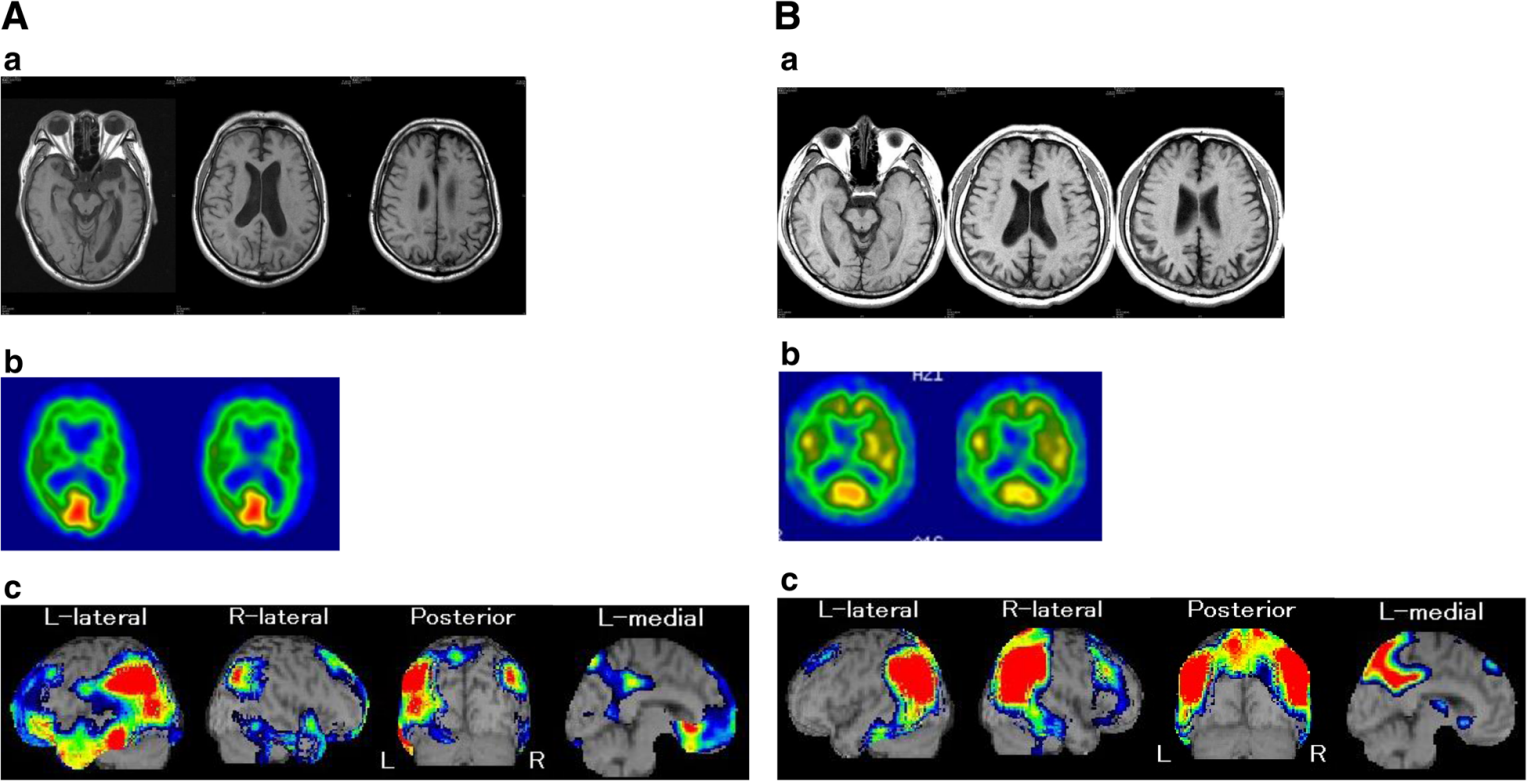
**a**. Brain magnetic resonance imaging and SPECT of Patient 1. *a* Brain magnetic resonance imaging showing atrophy in the left parietal region. *b* 99mTc-ethylcysteinate dimer single-photon emission computed tomography (Tc-99 m ECD SPECT) showing bilateral parietal hypoperfusion. *c* Tc-99 m ECD SPECT analyzed with eZIS showing relative hypoperfusion in the left temporoparietal region, which partly extended to the occipital lobe. **b**. Brain magnetic resonance imaging and SPECT of Patient 2. *a* Brain magnetic resonance imaging showing atrophy in bilateral parietal regions. *b* 99mTc-ethylcysteinate dimer single-photon emission computed tomography (Tc-99 m ECD SPECT) showing bilateral parietal hypoperfusion. *c* Tc-99 m ECD SPECT analyzed with eZIS showing relative hypoperfusion in bilateral temporoparietal regions, which partly extended to the occipital lobe.

At this time, he also showed apraxia. He became unable to use nail clippers and tried to cut his nails with scissors instead. He clearly understood what the nail clippers were used for but he was not sure how to use them. No ideomotor apraxia was observed with either hand when asked to imitate the examiner’s gesture. He showed no visual agnosia or Bálint syndrome in the visual perception test for agnosia [13]. He showed no visuoconstruction disorders when copying a cube. Affect and concern with hygiene were preserved, and his personality did not change. His score on the Clinical Dementia Rating scale [14] remained at 0.5 for the initial 2 years of his illness. Brain magnetic resonance imaging demonstrated atrophy in the left temporoparietal cortices (Fig. 1a). We evaluated the patient with Tc-99 m ECD SPECT [15] (Fig. 1a), which demonstrated relative hypoperfusion mainly in the left temporoparietal areas, which partly extended to the occipital lobe. The main affected areas were slightly posterior and somewhat distant from the perisylvian area, the main area usually affected by lvPPA [2, 3]. The affected area also differed from that of svPPA in which the main affected area is typically the temporal pole [1].

At age 67, he put pesticide on a wound after he was bitten, confusing it with an antipruritic drug. On another occasion, he put denture cleaner on his garden plants. He also began to present with episodic memory deficits and tended to forget his schedule or what he had said.

His aphasia gradually worsened. At age 70, although his repetition of short sentences remained intact, he often could not understand what simple words meant. His comprehension of single words fell to 60 %, and his comprehension of command sentences became 0 % on the SLTA. His naming ability fell to 0 %. He was no longer able to read or write, including his own name and address. He was also unable to recognize tools used in everyday life. Eventually, he began to often eat non-edible objects such as sponges. He was admitted to a nursing home. The clinical features of this case most likely suggest Alzheimer’s disease.

### Patient 1 summary

This patient presented with transcortical sensory aphasia and ideational apraxia that insidiously worsened. Agraphia and acalculia were also observed. Episodic memory deficits became apparent 2 years after the onset of symptoms. Ideational apraxia progressed into conceptual apraxia. Hypoperfusion was found mainly in the left temporoparietal area.

### Patient 2

A 50-year-old right-handed man with 16 years of education began to experience progressive word-finding difficulty. He had been a system engineer but began making mistakes at his work because he was unable to understand exactly what his manager told him. He had no previous medical or psychiatric problems. His older sister had been clinically diagnosed with Alzheimer’s disease at age 60. He was referred to our hospital for evaluation of cognitive function at age 52, 2 years after the onset of cognitive decline. His laboratory results were normal. Neurological examination was normal with no pyramidal, extra-pyramidal nor cerebellar signs. Neuropsychological examination (Table 1) showed word-finding difficulty but no phonological paraphasias. Grammar, articulation, prosody, and repetition were also preserved. Conversational speech was hesitant, with word-finding pauses. The maximum length of sentence repetition was six phrases. Non-word repetition was preserved. His digit span was a maximum of six digits forward and four digits backward. Comprehension of single words was intact, whereas comprehension of complex sentences was impaired. For example, although he was able to comprehend simple oral sentences such as “Touch your ankle”, he was unable to comprehend complex oral sentences such as “Touch the book on the table with the pen first, then give me the pen”. His correct naming was 65 % on the SLTA [7]. Although his comprehension of single words remained 100 %, comprehension of command sentences was 50 %. Taken together, his aphasia was categorized as transcortical sensory aphasia with preserved repetition and word meaning. He also made mistakes in writing, in particular, in Kanji, or Chinese characters. Although he was a university graduate, he could not write some Kanjis, which are taught in lower grades of primary school. Although reading was relatively preserved compared with writing, comprehension of written complex sentences was also impaired as was oral comprehension. He also showed acalculia, in particular when carrying and borrowing were needed. Reading comprehension of complex sentence commands was only 20 % on SLTA. Written naming in Kanji was 20 % and dictation of short sentences was 0 %. His correct calculation was 10 %. In addition, he was no longer able to read an analog clock. While driving, he became unsure of how to operate windshield wipers. Although he showed no visual agnosia or any signs of Bálint syndrome in the visual perception test for agnosia [13], he showed visuoconstruction disorders when copying a cube. Affect and concern with hygiene were preserved, and his personality did not change. His score on the Clinical Dementia Rating scale [14] remained at 0.5 for the initial 2 years of his illness. Brain magnetic resonance imaging demonstrated atrophy in the bilateral parietal cortices (Fig. 1b). We also evaluated the patient with Tc-99 m ECD SPECT (Fig. 1b), which demonstrated relative hypoperfusion in the bilateral temporoparietal areas, which partly extended to the occipital lobe.

At age 53, he began to complain of difficulty using electrical appliances such as vending machines and the remote control for an air conditioner. He clearly understood what these appliances are used for but was unsure how to operate them. No ideomotor apraxia was observed with either hand when asked to imitate the examiner’s gesture. He also showed dorsal simultanagnosia, one of the three sign of Bálint syndrome in the visual perception test for agnosia [13]. At age 54, he began to present with episodic memory deficits and tended to forget his schedule or what he had said. He could not copy Kanjis, nor could he even trace them. At age 55, he was no longer able to fasten his seatbelt. He showed apraxia of dressing and often put clothes on backwards or upside down. His aphasia gradually worsened.

At age 56, although his repetition of short sentences remained intact, he often could not understand what words meant. His comprehension of single words fell to 70 %, and his comprehension of command sentences became 0 % on the SLTA. His naming ability fell to 0 % on the SLTA. He became unable to flush the toilet in his house. When asked to use a comb, a toothbrush, or a fan, he was not sure how to use them, although he clearly understood what these tools are and what they are used for. He could not put his legs into his own horigotatsu, a Japanese foot warmer consisting of a short-legged table over a hole in the floor with a foot warmer and a quilt over it. He occasionally began to get lost. At age 57, he was admitted to a nursing home. The clinical features of this case most likely suggest early-onset Alzheimer’s disease.

### Patient 2 summary

This patient presented with transcortical sensory aphasia and ideational apraxia as well as visuospatial deficits, which gradually worsened. Episodic memory deficits became apparent 4 years after the onset of symptoms. Hypoperfusion was demonstrated mainly in the bilateral temporoparietal areas.

## Discussion

Two cases of progressive transcortical sensory aphasia and progressive apraxia were documented. Both cases had some features of posterior cortical atrophy, such as agraphia, acalculia, and visuospatial deficits. Hypoperfusion was found mainly in the left temporoparietal area. Although our cases lack in CSF findings and PIB scan, the clinical features of this case most likely suggest Alzheimer’s disease. These patients first began to show cognitive decline at age 50 and age 65, similar to patients with svPPA, posterior cortical atrophy, and primary progressive apraxia, who tend to be young with a typical age of onset in the 50s or early 60s [2–9]. The insidiously progressive perturbation of language usage suggests PPA. However, these patients do not fulfill criteria for PPA because the prominent clinical features include not only aphasia but also apraxia. In addition, they cannot be clearly categorized into any of the three recognized PPA subtypes, namely naPPA, svPPA, and lvPPA. Linguistic features of svPPA somewhat resemble progressive transcortical sensory aphasia in terms of preserved repetition and anomia. However, svPPA differs from our cases of progressive transcortical sensory aphasia as follows. First, patients with svPPA have substantial word meaning deficits in the early stages [1], whereas those with progressive transcortical sensory aphasia develop word meaning deficits in the later stages. Second, unlike our patients, patients with svPPA frequently have or develop social behavioral problems, e.g., a personality change, as a consequence of frontotemporal lobar degeneration [1]. Third, in general, patients with svPPA do not present with apraxia [1], whereas our cases involved apraxia that began at the initial stages. Fourth, the areas affected most frequently in svPPA patients are the temporal poles [1], whereas the areas affected in our cases were mainly the posterior part of the temporal lobe. Finally, the neuropathological diagnoses may be different.

Before the concept of PPA was developed, previous studies had shown that transcortical sensory aphasia and Wernicke’s aphasia [16–18] were sometimes present in patients with Alzheimer’s disease, in which the main affected areas are the posterior cortices. The anatomical areas of hypoperfusion described here are similar to those of lvPPA in which the main areas with atrophy are the left temporoparietal area or its underlying white matter surrounding the posterior perisylvian sulcus. Patients with lesions in these areas present with phonological deficits [19], a hallmark of both Wernicke’s/conduction aphasia and lvPPA. However, the lesions in our cases were slightly posterior and somewhat distant from the perisylvian sulcus. Unlike lvPPA patients, our cases did not present with phonological deficits. A slight difference in the main affected areas may reflect differences in linguistic features. These linguistic characteristics resemble those of Wicklund’s patients with posterior cortical atrophy [10] in whom aphasia was characterized by fluent output with normal grammar and syntax, anomia without loss of word meaning, and relatively spared repetition. The characteristics of our cases and previous studies [10, 11], in particular transcortical sensory aphasia along with some forms of posterior symptoms (agraphia, acalculia, and visuospatial deficits), may reflect the spectrum between lvPPA and posterior cortical atrophy. In fact, the first five described cases of posterior cortical atrophy [20] with higher visual dysfunction were reported to have developed transcortical sensory aphasia in later stages. According to Kas et al. [21], 21 of 39 patients (54 %) with posterior cortical atrophy developed aphasia at a mean disease duration of 3.8 ± 2.1 years, although they did not mention what type of aphasia these patients had. These studies indicate that aphasia—in particular transcortical sensory aphasia—develops in patients with posterior cortical atrophy in later stages. Leyton et al. [22] found that patients with lvPPA develop visuospatial dysfunction more rapidly than those with svPPA. Considering these studies, the distinction between lvPPA and posterior cortical atrophy may be less clear than previously thought. From a biological viewpoint, Migliaccio et al. [23] indicated that these two forms of Alzheimer’s disease show largely overlapping anatomical and biological features, i.e., a high frequency of the APOEε4 allele, suggesting that these two syndromes represent the spectrum of clinical manifestation of the non-typical form of Alzheimer’s disease that presents at an early age. Their view is compatible with the similarity between the two clinical manifestations lvPPA and posterior cortical atrophy.

Apraxia has not been thoroughly investigated in patients with lvPPA and posterior cortical atrophy, and few articles have been published on this topic. Kas et al. [21] documented that ideomotor apraxia was found in 37 of 39 patients with posterior cortical atrophy. Given their visuospatial deficits and the impact on imitation of the examiner’s gesture, the presence of ideomotor apraxia, which involves imitation, in patients with posterior cortical atrophy is not surprising. Teichmann et al. [24] found that lvPPA due to probable Alzheimer’s pathology involves ideomotor apraxia along with extensive language/cognitive disorders. However, this was not the case with our patients who had ideational/conceptual apraxia but not ideomotor apraxia. At the initial stage, our patients had ideational apraxia, in which they did not understand how to use tools, although they clearly understood what those tools were used for. Ideational apraxia in patient 1 developed into conceptual apraxia, a disturbance in action-semantic representations, in which he showed semantic parapraxia such as putting denture cleaner on his garden plants. It might be reasonable that our patients had ideational/conceptual apraxia because the main areas showing atrophy involved the parietal cortex, which is associated with various types of apraxia due to non-degenerative [25] and degenerative disorders [7–9]. However, previous studies on primary progressive apraxia have mainly focused on limb-kinetic apraxia, which is usually observed in patients with corticobasal syndrome. Conceptual apraxia, which was noted in patient 1, has frequently been documented in patients with Alzheimer’s disease [26–29]. Iizuka et al. [30] found that a patient with posterior cortical atrophy developed ideational apraxia along with other visuospatial deficits. We also reported three cases with lvPPA who presented with several types of apraxia including ideational/conceptual apraxia [31]. Little attention has apparently been paid to ideational/conceptual apraxia in lvPPA and posterior cortical atrophy. Considering the impact of apraxia on activities of daily living, assessing ideational/conceptual apraxia in patients with lvPPA and posterior cortical atrophy is important, especially in those with prominent parietal cortex atrophy.

## Conclusions

Although our cases lack in CSF findings and PIB scan, these two cases and the other reported cases [10, 11] might suggest the existence of a subgroup of patients presenting with transcortical sensory aphasia, apraxia, and posterior symptoms (acalculia, agraphia, and visuospatial deficits) in the setting of Alzheimer’s disease. This subgroup may reflect the spectrum of clinical manifestations between lvPPA and posterior cortical atrophy.

## Consent

Written informed consent was obtained from the spouse of each patient because the patients were incapable of giving consent due to their aphasia and severe cognitive impairment.

## Abbreviations

naPPA: non-fluent/agrammatic primary progressive aphasia
svPPA: semantic variant of PPA
lvPPA: logopenic variant of PPA
Tc-99 m ECD SPECT: 99mTc-ethylcysteinate dimer single-photon emission computed tomography
SLTA: standard language test of aphasia
